# Surface Defect Detection for Automated Tape Laying and Winding Based on Improved YOLOv5

**DOI:** 10.3390/ma16155291

**Published:** 2023-07-27

**Authors:** Liwei Wen, Shihao Li, Jiajun Ren

**Affiliations:** 1 College of Material Science and Technology, Nanjing University of Aeronautics and Astronautics, Nanjing 210016, China; lishihao@nuaa.edu.cn; 2 Haiying Aerospace Materials Research Institute (Suzhou) Co., Ltd., Suzhou 215100, China; renjiajun@nuaa.edu.cn

**Keywords:** automated tape laying and winding, surface defect detection, YOLOv5

## Abstract

To address the issues of low detection accuracy, slow detection speed, high missed detection rate, and high false detection rate in the detection of surface defects on pre-impregnated composite materials during the automated tape laying and winding process, an improved YOLOv5 (You Only Look Once version 5) algorithm model was proposed to achieve the high-precision, real-time detection of surface defects. By leveraging this improvement, the necessity for frequent manual interventions, inspection interventions, and subsequent rework during the automated lay-up process of composite materials can be significantly reduced. Firstly, to improve the detection accuracy, an attention mechanism called “CA (coordinate attention)” was introduced to enhance the feature extraction ability, and a Separate CA structure was used to improve the detection speed. Secondly, we used an improved loss function “SIoU (SCYLLA-Intersection over Union) loss” to replace the original “CIoU (Complete-Intersection over Union) loss”, which introduced an angle loss as a penalty term to consider the directional factor and improve the stability of the target box regression. Finally, Soft-SIoU-NMS was used to replace the original NMS (non-maximum suppression) of YOLOv5 to improve the detection of overlapping defects. The results showed that the improved model had a good detection performance for surface defects on pre-impregnated composite materials during the automated tape laying and winding process. The FPS (frames per second) increased from 66.7 to 72.1, and the mAP (mean average precision) of the test set increased from 92.6% to 97.2%. These improvements ensured that the detection accuracy, as measured by the mAP, surpassed 95%, while maintaining a detection speed of over 70 FPS, thereby meeting the requirements for real-time online detection.

## 1. Introduction

As advanced composite materials are widely applied in fields such as aerospace, the automotive industry, and medicine, related automation technologies such as composite laying and winding are also constantly developing and maturing [1,2,3]. Due to the processes of automated tape laying and winding, such as tension control, laying trajectory, initial positioning, and the adhesive properties of the pre-impregnated material, various defects can occur during the automated tape laying and winding process. These defects can compromise the structural integrity of the components and impact their performance. Inclusion defects arise when the recovery device fails to separate the pre-impregnated tape and the backing film due to excessive viscosity. As a result, both the tape and film are laid on the mold together during the automated tape laying and winding process. Typically, clearance defects are accompanied by overlap defects, and two types of clearance/overlap defects can be observed in composite component manufacturing: inconsistent widths of the pre-impregnated tape during production and tape misalignment caused by issues with the laying trajectory. Fold defects can be attributed to three main factors. Firstly, folds may originate from the pre-impregnated tape itself during its production. Secondly, failure of the bond strength between the pre-impregnated material and the mold can lead to folding. Lastly, variations in the curvature radius along the laying trajectory result in different curvatures on each side of the pre-impregnated tape. As the lay-up machine undergoes turning, tension on one side and compression on the other side contribute to the formation of folds through the combined effects of these forces.

Currently, most defect detection during automated laying and winding processes, as well as the final product inspection, still relies on manual inspection. The full automation of composite material manufacturing has not been achieved yet, and the bottleneck lies in the essential defect detection process. In order to meet the high-performance requirements of composite components, the machine must stop after each layer is laid down for visual inspection and confirmation by the inspection personnel. Moreover, frequent rework is required to meet the quality assurance and requirements. At the same time, manual inspection has many drawbacks, such as high labor costs, a slow inspection speed, a low efficiency, and a tendency to miss defects. Additionally, manual inspection is subject to physiological and psychological factors, and the results can be greatly influenced by subjective factors. Different operators may have different opinions and understandings of defects, resulting in inconsistent inspection results and product quality. For a large-scale composite component that requires hundreds of layers, inspection and rework have a huge impact on production speed.

In the field of automated surface defect detection for composite materials, some non-manual detection methods have been developed over time; however, they still have limitations. Initially, specific measurement devices are used to obtain feature change maps (with simplistic color information), which are then subjected to detection algorithms for further analysis. Christopher Sacco et al. [4] used a profilometer to measure the point cloud of the layer surface and transformed it into a grayscale image. They designed a machine learning algorithm based on fully convolutional neural networks to classify defects in the grayscale image. However, point cloud data are typically voluminous and require complex algorithms and high-performance computing devices, making real-time detection challenging. Carsten Schmidt et al. [5] developed an AFP (Automated Fiber Placement) defect detection system based on infrared thermography. A heating lamp was used to heat the surface of the pre-impregnated material in front of the pressure roller, while a thermal imager was placed behind the pressure roller to obtain a thermal image of the surface. A simple CNN (Convolutional Neural Network) network was then utilized to analyze the thermal image for defect classification. However, due to the non-uniform distribution of the heat, the temperature of the pre-impregnated material near the heat source was higher than that farther away, resulting in different temperature field variations. Moreover, the temperature data were highly influenced by temperature, resulting in a low detection accuracy. Sebastian Zambala et al. [6] utilized a laser triangulation sensor to scan the surface of the laminate to obtain a height feature image. When there were defects on the surface, the height feature image would differ, and then the U-Nets algorithm [7] was used to segment the height feature image to achieve defect detection. This system could only detect flat composite components, and could detect changes on the mold surface due to height variations.

In recent years, deep learning has developed rapidly, and its excellent performance in object detection has provided a new approach for research on the online detection of surface defects in automated tape laying and winding. Common object detection algorithms in deep learning include Faster R-CNN (Faster Region-based Convolutional Neural Network), YOLO, SSD (Single Shot MultiBox Detector), and Transformer, among others [8,9,10]. YOLO, proposed by Joseph Redmon in 2016, has attracted attention, and YOLOv3, proposed by him in 2018, is favored by the industry due to its excellent detection speed and accuracy. In April 2020, Alexey Bochkovskiy combined the recent outstanding ideas in the field of computer vision with YOLOv3 to form YOLOv4 [11,12], which had a higher speed and accuracy than the other outstanding detection algorithms at that time. In June 2020, Glenn Jocher released YOLOv5, which further improved the detection speed and accuracy, and significantly improved the inference speed. However, compared to other industrial fields that have successfully applied deep learning [11,13], there are currently two reasons why deep learning has not been applied to detect the surface defects in composite materials during automated laying and winding. First, there are a lack of high-quality datasets for surface defects in composite materials during automated laying and winding. Second, a large number of small defects are generated during the automated laying and winding process, and the current object detection algorithms have a relatively low detection accuracy for small targets.

To address these issues, this paper proposes an improved object detection algorithm based on YOLOv5 to achieve the end-to-end detection of the defects generated during the automated laying and winding process of composite materials, directly processing images or videos of the laying and winding process. The main contributions of this paper are as follows:

(1) The CA attention mechanism has been improved in the embedded Separate CA structure, which not only achieves long-range dependence in the spatial direction, but also enhances the positional information and improves the ability to extract features. Additionally, the use of interval embedding further enhances the detection speed.
(2) A new SIoU_loss regression box loss function has been proposed to replace the original CioU_loss loss function, introducing considerations for the matching direction and using the angle loss as a penalty term. This further accelerates the regression speed of the bounding box and improves the detection accuracy, especially for small objects.
(3) Based on the proposed SIoU_loss regression box loss function in this paper and combined with the Soft-NMS regression box filtering method, a new non-maximum suppression method called Soft-SIoU-NMS has been proposed for the post-processing of the model. By using a more gentle pre-selection box removal method, redundant boxes are removed while retaining more effective boxes, which improves the detection accuracy for overlapping coverage defects.

## 2. Detection Principle of YOLOv5

The YOLO models (YOLOv1–YOLOv5) are one-stage detection algorithms that can achieve end-to-end detection. They treat object detection as a regression problem and do not use the sliding window method. Instead, the images are directly input for training, allowing for a clear distinction between the background and the objects being detected, greatly improving the detection speed. Among them, YOLOv5 is currently the fastest and most accurate model, with great application prospects. YOLOv5 is an improvement on YOLOv4, with optimization in four parts: input end, backbone network, neck layer, and output end, further improving the detection accuracy and speed [13,14].

**Figure 1:**
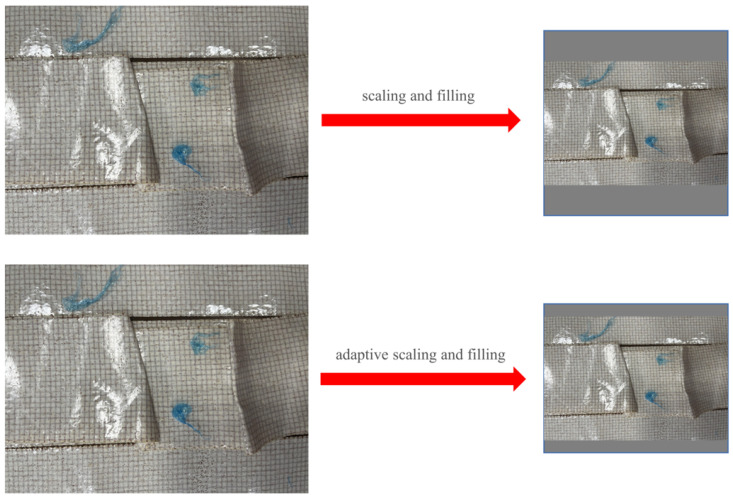
Adaptive scaling with padding.

The input stage refers to the component of the algorithm responsible for receiving images as inputs. In the case of YOLOv5, it accepts images as inputs to facilitate object detection. On the input side, YOLOv5 uses the same Mosaic data augmentation as YOLOv4 [15,16], but adds adaptive anchor box calculation and adaptive image scaling. YOLOv5 incorporates anchor box generation into the code so that it can calculate the most suitable aspect ratio anchor boxes for the current dataset. In terms of the images of surface defects in the composite material winding components, the size of these images cannot be guaranteed to be consistent during the shooting process, but it is necessary to ensure consistency in the inputs to the network training. The most common practice is to resize the images to a uniform size before training the network. Upon scaling, due to the varying aspect ratios, gray borders are employed for padding. However, conventional resizing with fixed input dimensions may result in excessive gray borders, leading to cluttered information and a compromised speed. Hence, an adaptive image scaling approach is adopted to adjust the size of these gray borders. The main steps involve calculating the scaling ratio as r = min(640/w, 640/h), resizing the dimensions accordingly based on this scaling ratio, and padding the other dimension to be divisible by the stride (e.g., 32). As depicted in Figure 1, this operation significantly reduces the presence of gray borders. Simply scaling the image and forcefully padding it may lead to a loss of image information and an ineffective utilization of the receptive field information.

The backbone network is a combination of convolutional and pooling layers designed to extract features from the input images. In YOLOv5, a backbone network is utilized to extract both low-level and high-level features from the input images, enabling a better understanding of the image content. In the backbone network, a convolutional layer with a 6 × 6 kernel is used instead of the Focus structure. On the one hand, this replaces the original three convolutional layers and improves the computational speed, while maintaining the same receptive field. On the other hand, replacing the Focus structure reduces the number of Concat operations and lowers the memory overhead.

**Figure 2:**
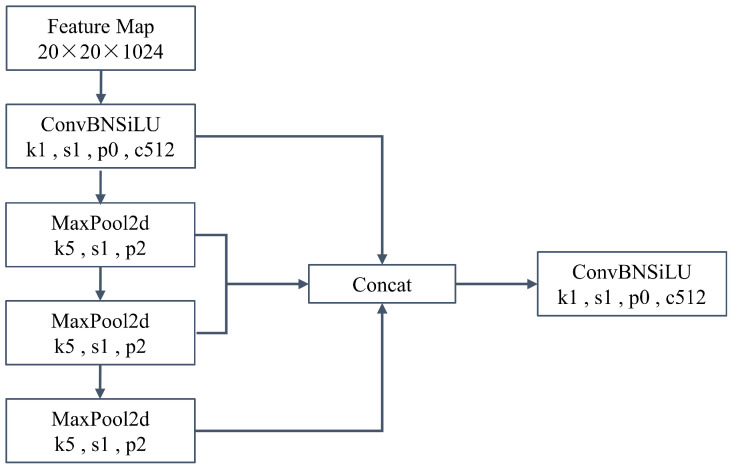
Sequential Passing of SPPF Structure Through Multiple 5 × 5 MaxPool Layers.

The neck layer is the component located between the backbone network and the output layer. It plays a crucial role in connecting the backbone network and the output layer, while performing feature fusion and dimensionality reduction. In YOLOv5, the neck layer aids in transforming the features extracted from the backbone network into a format suitable for object detection. The neck also consists of the FPN (Feature Pyramid Network) + PAN (Path Aggregation Network) [17] structure, but with the addition of the CSP2 (Cross Stage Paritial Network2) structure to enhance the network’s feature fusion ability. In this section, the SPP (Spatial Pyramid Pooling) structure is replaced with the SPPF (Spatial Pyramid Pooling—Fast) structure, which involves passing the input through multiple 5 × 5 max pooling layers in series, as shown in Figure 2. Serially applying two 5 × 5 max pooling layers is equivalent to applying one 9 × 9 max pooling layer, and applying three in series is equivalent to one 13 × 13 max pooling layer. This approach not only achieves the same effect as the SPP, but also further improves the computational speed.

The output layer is the final layer of the network responsible for generating the results of the object detection. In YOLOv5, the output layer generates predicted bounding boxes and corresponding class confidences, which determine the presence of objects in the image and their respective locations. In the output layer of YOLOv5, the CIoU_Loss function is utilized as the bounding box loss function, while the NMS (non-maximum suppression) method is employed for handling overlapping objects.

## 3. The Improvement of the Model Based on YOLOv5

### 3.1. Separate CA-YOLOv5 Network Model

The attention mechanism [18] is a biomimetic model that imitates the human ability to focus only on important parts. In this section, the attention mechanism is introduced to mimic the surface defects that occur during the composite material laying process observed by human eyes. This enables the detection network to quickly focus on the defect target and find the region of interest in the image, thereby improving the detection accuracy. Additionally, the color and shape features of the inclusion defects and gap defects in composite materials are more distinct from the background, and the introduction of the attention mechanism can more effectively extract these features.

**Figure 3:**
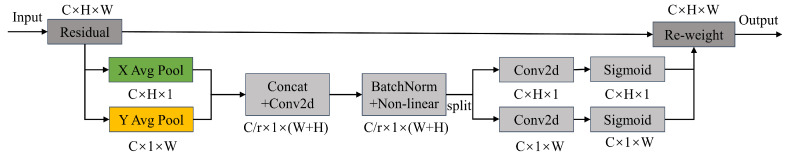
CA attention mechanism.

As shown in Figure 3, the CA [19] attention mechanism not only achieves long-term dependency in the spatial direction, but also takes into account the position information, enhancing the expression of the feature position information. Compared to CBAM (Convolutional Block Attention Module) [20], it combines spatial attention [21] and Channel attention [22].

The CA module decomposes global pooling with the following formula:

(1) $$Z_{c}=\frac{1}{H\times W}\sum_{i=1}^{H}\sum_{j=1}^{w}x_{c}\left(i,j\right)$$

When the height is h and the channel number is *c*, this can be represented by the following formula:

(2) $$z_{c}^{h}\left(h\right)=\frac{1}{W}\sum_{0\leq i<W}x_{c}\left(h,i\right)$$

When the width is w and the channel number is *c*, this can be represented by the following formula:

(3) $$z_{c}^{w}\left(w\right)=\frac{1}{H}\sum_{0\leq j<H}x_{c}\left(j,w\right)$$

After obtaining the feature maps separately on the width and height, they are concatenated together and then reduced in dimensionality through a 1 × 1 convolution. The resulting feature map is normalized and passed through the sigmoid activation function to obtain a feature map of 1 × (W+H) × (C/r).

(4) $$f=\delta \left(F_{1}\left(z^{h},z^{w}\right)\right)$$

In the above equation, δ represents the non-linear activation function and [*,*] denotes the spatial concatenation operation. After completing the aforementioned operations, the feature map undergoes a 1 × 1 convolution along both the height and width to obtain feature maps $F_{h}$ and $F_{w}$, both with the same number of channels as the original. These two feature maps undergo a transformation using a 1 × 1 convolution, resulting in the attention weights $g^{h}$ and $g^{w}$, defined as follows:

(5) $$g^{h}=\sigma \left(F_{h}\left(f^{h}\right)\right)$$

(6) $$g^{w}=\sigma \left(F_{w}\left(f^{w}\right)\right)$$

In the above equation, σ represents the sigmoid activation function. After obtaining the attention weights, a multiplication operation is performed on the feature map to obtain the weighting. The two feature maps with specific spatial information obtained through the above method complement each other. Ultimately, an attention-weighted feature map is obtained, defined as follows:

(7) $$y_{c}\left(i,j\right)=x_{c}\left(i,j\right)\times g_{c}^{h}\left(i\right)\times g_{c}^{w}\left(j\right)$$

**Figure 4:**
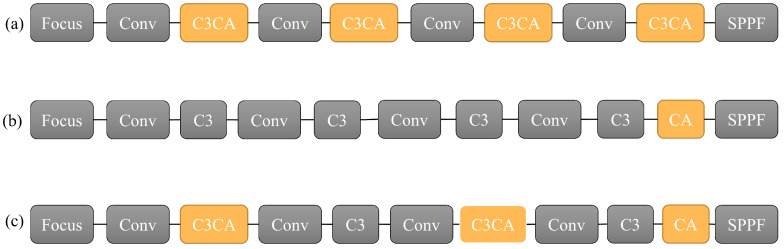
Comparison between common embedding methods of CA modules and the embedding method in this article. (**a**) C3CA structure (**b**) CA structure (**c**) Separate CA structure.

The CA module is a plug-and-play module that can be embedded behind any feature extraction module in the YOLOv5 structure. Commonly, the CA module is integrated into the backbone network, as shown in Figure 4.

Figure 4a shows the C3CA structure, which embeds the CA attention module into the C3 module and applies the attention mechanism to each generated feature map. Figure 4b shows the CA structure, which embeds the CA attention module after the last C3 module and applies the attention mechanism to the deeper feature maps. Figure 4c proposes a new embedded Separate CA structure, which is an interval insertion structure. Firstly, the image undergoes the attention mechanism during the initial feature extraction stage, and the feature focus can enhance the positional information of the feature map. Then, the feature map with a small receptive field that contains positional information is subjected to another feature extraction, and after another round of the CA attention mechanism, a feature map with a larger receptive field and richer positional information is obtained. Lastly, a CA attention mechanism is added to the end to obtain a feature map with a larger receptive field and directional perception.

Compared to the CA structure, the Separate CA structure proposed in this paper adds the attention mechanism in the middle of the feature extraction process, greatly enhancing the feature extraction of the feature map, integrating positional information into the feature map, and obtaining more semantic information. Compared to the C3CA structure, the Separate CA structure reduces one attention module, not only reducing the complexity of the model, facilitating a speed improvement in the model, but also enriching the feature information by adding a separate CA module to the end of the feature map, which better improves the detection accuracy.

### 3.2. Regression Box Loss Function Improvement

In YOLOv5, the CIoU loss function [23] was originally used as the bounding box localization loss, which optimized the overlap area, distance between the center points, and aspect ratio between the predicted and ground truth boxes. The expression is as follows:

(8) $$\begin{array}{c} CIoU=IoU-\left(\frac{P^{2}\left(B,A\right)}{C^{2}}+\alpha v\right) \end{array}$$

(9) $$\begin{array}{c} v=\frac{4}{\pi^{2}}{\left(\arctan\frac{w^{A}}{h^{A}}-\arctan\frac{w^{B}}{h^{B}}\right)}^{2} \end{array}$$

(10) $$\begin{array}{c} \alpha =\frac{v}{\left(1\;-\;IoU\right)\;+\;v} \end{array}$$

When $w^{B}$ = ${kw}^{A}$ and $h^{B}$ = ${kh}^{A}$, and v takes a value of 0, the value of αv is 0, which renders the optimization based on the aspect ratio ineffective. This ultimately results in the predicted box being unable to fit the ground truth box. By taking the partial derivative of Formula (9), we obtain the following equation:

(11) $$\begin{array}{c} \frac{\partial_{v}}{{\partial w}^{B}}=\frac{8}{\pi }(\arctan\frac{w^{7gt}}{h^{gt}}-\arctan\frac{w^{B}}{h^{B}})\times \frac{h^{B}}{{\left(w^{B}\right)}^{2}+{\left(h^{B}\right)}^{2}} \end{array}$$

(12) $$\begin{array}{c} \frac{\partial_{v}}{\partial h^{B}}=-\frac{8}{\pi }(\arctan\frac{w^{10gt}}{h^{gt}}-\arctan\frac{w^{B}}{h^{B}})\times \frac{w^{B}}{{\left(w^{B}\right)}^{2}+{\left(h^{B}\right)}^{2}} \end{array}$$

As shown in the equation above, $h^{B}$ and $w^{B}$ cannot both decrease at the same time during the regression. For composite materials with laying and winding surface defects, the preset anchor box size often exceeds the size of the defect, leading to situations where two boxes may overlap during training. Additionally, except for CIoU (Complete-Intersection over Union), other loss functions such as GIoU (Generalized-Intersection over Union) [24] and DIoU (Distance-Intersection over Union) [25], etc., have not considered the directionality between the predicted and ground truth boxes. Specifically, when the center points of the predicted and ground truth boxes are close, these loss functions will all degrade to IoU (Intersection over Union) [26], resulting in a slow convergence. To address this issue, the SIoU (SCYLLA-Intersection over Union) loss function is introduced to improve the localization regression loss by incorporating the vector angle between the predicted and ground truth boxes, redefining the relevant loss functions, which mainly include four cost functions:

(1) Angle cost

**Figure 5:**
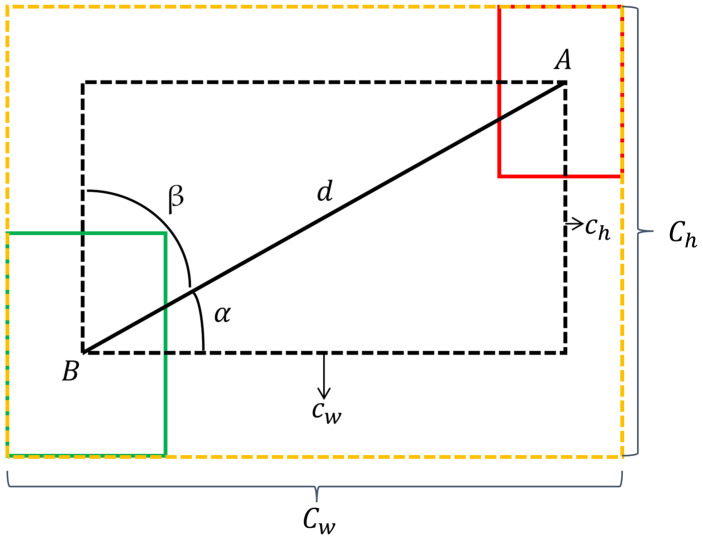
The Scheme for calculation of angle cost contribution into SIoU_loss.

As shown in Figure 5, the red rectangle A represents the ground truth box, the green rectangle B represents the predicted box, and the black dashed rectangle represents the rectangle formed by the centers of the ground truth and predicted boxes. d is the distance between the two centers, $c_{w}$ is the difference in width between the two centers, $c_{h}$ is the difference in height between the two centers, α is the angle between d and $c_{w}$, and β is the angle between d and $c_{h}$.

When α ≤ π/4, the convergence process will first minimize α. When π/4 < α ≤ π/2, the convergence process will minimize β. To achieve this, the following calculation formula is introduced:

(13) $$\begin{array}{c} \Lambda =1-2\times \sin^{2}\left(\arcsin\left(\frac{c_{h}}{d}\right)-\frac{\pi }{4}\right)=\cos\left(2\times \left(\arcsin\left(\frac{c_{h}}{d}\right)-\frac{\pi }{4}\right)\right) \end{array}$$

(14) $$\begin{array}{c} \frac{c_{h}}{d}=\sin\left(\alpha \right) \end{array}$$

(15) $$\begin{array}{c} d=\sqrt{{\left(A_{x}-B_{x}\right)}^{2}+{\left(A_{y}-B_{y}\right)}^{2}} \end{array}$$

(16) $$\begin{array}{c} c_{h}=max\left(A_{x},B_{x}\right)-min\left(A_{y},B_{y}\right) \end{array}$$

As shown in the above formula, ($A_{x}$, $A_{y}$) represents the center coordinate of the ground truth bounding box, while ($B_{x}$, $B_{y}$) represents the center coordinate of the predicted bounding box. When α = 0 or α = π/2, the predicted and ground truth bounding boxes are in a horizontal or vertical position, and the angle loss will be 0 in this case.

(2) Distance cost

Based on the angle loss defined above, the distance loss is redefined as follows:

(17) $$\begin{array}{c} \Delta =\sum_{t=x,y}\left(1-e^{-\gamma \rho_{t}}\right)=2-e^{-\gamma \rho_{x}}-e^{-\gamma \rho_{y}} \end{array}$$

(18) $$\begin{array}{c} \rho_{x}={\left(\frac{A_{x}-B_{x}}{C_{w}}\right)}^{2} \end{array}$$

(19) $$\begin{array}{c} \rho_{y}={\left(\frac{A_{y}-B_{y}}{C_{h}}\right)}^{2} \end{array}$$

(20) $$\begin{array}{c} \gamma =2-\Lambda \end{array}$$

As shown in Figure 5, the yellow dashed rectangle represents the minimum bounding box enclosing the ground truth and predicted boxes, where $C_{w}$ and $C_{h}$, respectively, denote the width and height of the minimum bounding box. It can be observed from the formula that, as α approaches 0, the contribution of the distance loss decreases. When α = 0 and γ = 2, it degenerates into the conventional distance loss. On the other hand, as α approaches π/4, the contribution of the distance loss increases.

(3) Shape cost

The definition of the shape loss is given by the following formula:

(21) $$\begin{array}{c} \Omega =\sum_{t=w,h}{\left(1-e^{-w_{t}}\right)}^{\theta }={\left(1-e^{-w_{w}}\right)}^{\theta }+{\left(1-e^{-w_{w}}\right)}^{\theta } \end{array}$$

(22) $$\begin{array}{c} w_{w}=\frac{\left|B_{w}-A_{w}|\right.}{max\left(B_{w},\;A_{w}\right)} \end{array}$$

(23) $$\begin{array}{c} w_{w}=\frac{\left|B_{h}-A_{h}|\right.}{max\left(B_{h},\;A_{h}\right)} \end{array}$$

where ($A_{w}$, $A_{h}$) and ($B_{w}$, $B_{h}$) denote the width and height of the ground truth and predicted boxes, respectively. The importance of the θ value cannot be overstated, as it regulates the extent to which attention is paid to the shape loss. When θ = 1, optimizing the shape loss would lead to the immediate optimization of the shape, thereby impeding its freedom of movement. To prevent an excessive focus on the shape loss and a decrease in the movement of the predicted boxes, a genetic algorithm is employed to calculate the value of θ for each dataset, which is determined to tend toward four. Consequently, the value of θ is restricted to the range [2,6].

(4) IoU cost

The IoU loss refers to the ratio of the intersection over union between the predicted bounding box and the ground truth bounding box.

In summary, the final definition of the SIoU loss function ($L_{SIoU}$) is given as follows:

(24) $$\begin{array}{c} L_{SIoU}=1-IoU+\frac{∆\;+\;\Omega }{2} \end{array}$$

By incorporating an angle penalty term into the SIoU loss function, the bounding box regression is further constrained, leading to an improved detection accuracy.

### 3.3. Post-Processing Method Improvements

#### 3.3.1. Post-Processing Method for YOLOv5

The bounding box regression filtering method used in YOLOv5 is NMS. After an image is input into the network, it is divided into S × S grids, and multiple bounding boxes with confidence levels are generated on each grid based on different object categories. The box with the highest score is selected from these boxes and the remaining boxes are discarded [27]. The specific process of this is as follows: (1) The regression boxes are divided according to the category and the background regression boxes are removed. (2) The regression boxes are sorted in descending order according to the classification confidence. (3) One type of target object is selected first. The regression box that ranks first in step 2 is removed from the input list and added to the output list. (4) The IoU value between all the regression boxes in the input list and the regression box with the highest confidence level is calculated. If the IoU value is greater than the threshold set, then the regression box is removed from the input list. (5) Steps 3–4 are repeated to distinguish all the regression boxes of this type of object. (6) Steps 2–5 are repeated to identify all the object categories and finally obtain the output list.

The expression for selecting the regression box in NMS is as follows:

(25) $$\begin{array}{c} S_{i}=\left\{\begin{array}{c} S_{i},IoU\left(A,B_{i}\right)<\varepsilon \\ 0,IoU\left(A,B_{i}\right)<\varepsilon \end{array}\right. \end{array}$$

In Formula (25), A represents the regression box with the highest confidence, B represents the remaining regression boxes, and ε is the set threshold. As can be seen from the above filtering process, the method of discarding all the boxes with an IoU greater than the set threshold may lead to inaccurate predictions. Firstly, this will cause two different types of targets to be removed due to overlapping regression boxes; for example, when a fold defect and a foreign object defect overlap, the foreign object defect may be covered by the fold defect and then be removed. Secondly, this operation can also filter out one of two defects of the same type due to occlusion, leading to missed detection, which greatly affects the detection performance. At the same time, NMS increases the complexity of the network calculation, which requires higher hardware requirements.

#### 3.3.2. Improving Non-Maximum Suppression Algorithm

Due to the suboptimal performance of NMS in regression box selection, this section proposes improvements based on two aspects.

The first improvement addresses the ‘all-or-nothing’ approach of NMS, which removes all the boxes with an IoU greater than the set threshold and is too aggressive. Therefore, the Soft-NMS approach is introduced, which uses a decay function f(x) with values between 0 and 1. Soft-NMS takes the highest-confidence regression box A and the predicted box $B_{i}$’s IoU value as inputs to the decay function. The confidence score of $B_{i}$ is then calculated by multiplying the decay function and the confidence score $S_{i}$, according to the following formula:

(26) $$\begin{array}{c} S_{i}=\left\{\begin{array}{c} S_{i}f\left(IoU\left(A,B_{i}\right)\right),IoU\left(A,B_{i}\right)\geq \varepsilon \\ S_{i},IoU\left(A,B_{i}\right)<\varepsilon \end{array}\right. \end{array}$$

The function f(x) uses a Gaussian decay function that has a better decay effect, as shown in Formula (27) below:

(27) $$f\left(IoU\left(A,B_{i}\right)\right)=e^{-\frac{{IoU\left(A,B_{i}\right)}^{2}}{\sigma }}$$

As described in the above formula, where σ is usually set to 0.5 and $f\left(IoU\left(A,B_{i}\right)\right)$ is guaranteed to be continuously valued in the range from 0 to 1, the Gaussian penalty function ensures that a higher IoU value during the selection process results in a greater penalty and lower score. For boxes with an originally low confidence, their existing confidence may fall below the confidence threshold after being penalized. For boxes with a high initial confidence, their confidence remains high after the penalty and they can be retained. In this way, the removal of overlapping object detection boxes can be reduced.

To address the limitation of using the IoU as the evaluation metric, which only considers the overlapping region between the bounding boxes and may not fully account for positional factors, resulting in missed detections, we propose using the SIoU as a substitute for the IoU to further improve the detection accuracy by incorporating angle factors into the optimization. The equation for calculating the confidence score is as follows:

(28) $$\begin{array}{c} S_{i}=\left\{\begin{array}{c} S_{i}f\left(SIoU\left(A,B_{i}\right)\right),SIoU\left(A,B_{i}\right)\geq \varepsilon \\ S_{i},SIoU\left(A,B_{i}\right)<\varepsilon \end{array}\right. \end{array}$$

(29) $$\begin{array}{c} f\left(SIoU\left(A,B_{i}\right)\right)=e^{-\frac{{SIoU\left(A,B_{i}\right)}^{2}}{\sigma }} \end{array}$$

## 4. Experimental Setup and Results Analysis

This section presents an exposition on the dataset, experimental framework, parameterization, and evaluation criteria adopted for conducting the experiments on the surface defect detection in composite materials. Subsequently, an analysis of the experimental results is discussed. To demonstrate and substantiate the effectiveness of the proposed method, a comparative study among five training models was conducted, with the models comprising the YOLOv5, YOLOv5 + SCA (Separate CA) with the integration of the CA attention mechanism, YOLOv5 + SIoU with an optimized regression box loss function, YOLOv5 + SSN (Soft-SioU-NMS) with an improved post-processing approach, and a new model that consolidates all three improved methods proposed in this manuscript.

### 4.1. Dataset

**Figure 6:**
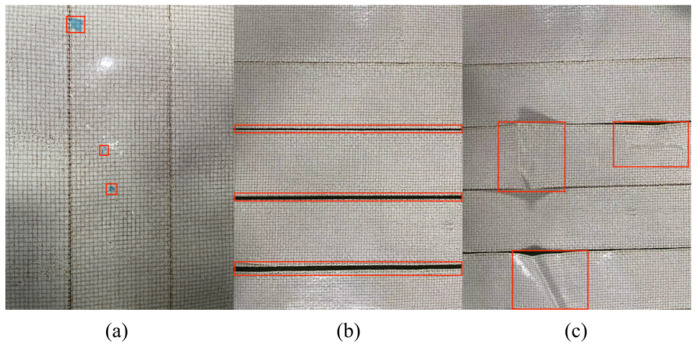
Partial Dataset (**a**) Inclusion (**b**) Clearance, and (**c**) Fold.

Currently, there is no systematically compiled dataset of surface defects in composite material automated tape laying and winding. This dataset combines defects captured by industrial cameras and lenses during actual automated tape laying and winding processes, as well as defects obtained through artificial, simulated automated tape laying and winding, in order to obtain a larger and more comprehensive defect dataset. The pre-impregnated material used in this dataset is a new type of lightweight phenolic resin ablation material, produced by Hubei Sanjiang Aerospace Hongyang Electromechanical Co., Ltd. In this study, a total of 3200 images from the defect dataset were selected. The defect types were categorized as inclusions, clearances, and folds, as shown in Figure 6. The image annotation software LabelImg (https://github.com/HumanSignal/labelImg) was used to label each image in the dataset and generate XML files. These files contained information about the category, dimensions, and position of the target defects.

### 4.2. Experimental Environment and Parameter Configuration

The experimental environment and resource configurations are essential prerequisites for network model training. For this experiment, both the software and hardware configurations are shown in Table 1.

The experiment was conducted on a Windows 10 × 64 operating system, using Pycharm as the IDE, and the overall software platform consisted of Windows 10, Pytorch 1.10.1, Python 3.9, CUDA 10.2.89, cuDNN 8.3.3, and Pycharm 2020.1. The hardware configuration included an Intel(R) Core(TM) i7-10875H 2.30 GHz processor, 32 GB of memory, and an NVIDIA GeForce GTX 1650 Ti.

The defective image was input with a size of 640 × 640 (due to YOLOv5 using adaptive scaling and padding technology at the input end, the actual preprocessed size may not have been exactly 640 × 640 on both sides. Only one side was 640, and the padding size of the other side needed to be calculated). Considering the size of the dataset, the number of general iterations, and the training results of Section 3 for the model, the number of iterations in this section was set to 300 times. Taking into account the experimental software and hardware configuration, including the video memory and other reasons, the batch size was set to 3. At the same time, the initial learning rate and weight decay coefficient were set to 0.01 and 0.0005, respectively. The specific parameter settings are shown in Table 2.

### 4.3. Evaluation Indicators

In this experiment, the evaluation criteria included the recall, AP, FPS, mAP, and other indicators. Recall is used to describe the proportion of correctly detected defects to the total number of defects that should be correctly detected. mAP and AP are both indicators of the detection performance. mAP reflects the overall performance of the entire object detection network for all the categories in the dataset, while AP reflects the comprehensive performance of the individual category detection in the dataset. FPS represents the number of images that the detector can process per second.

### 4.4. Experimental Results and Analysis

#### 4.4.1. Training Results and Analysis

**Figure 7:**
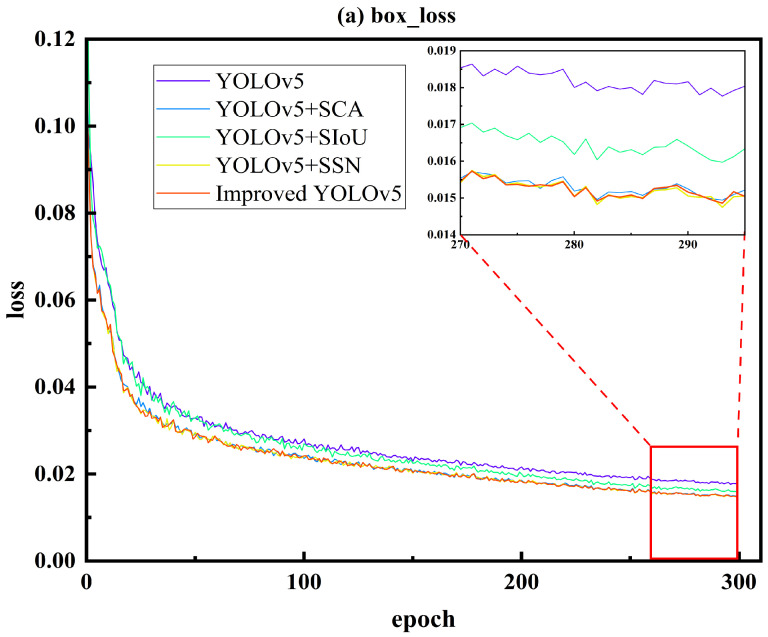

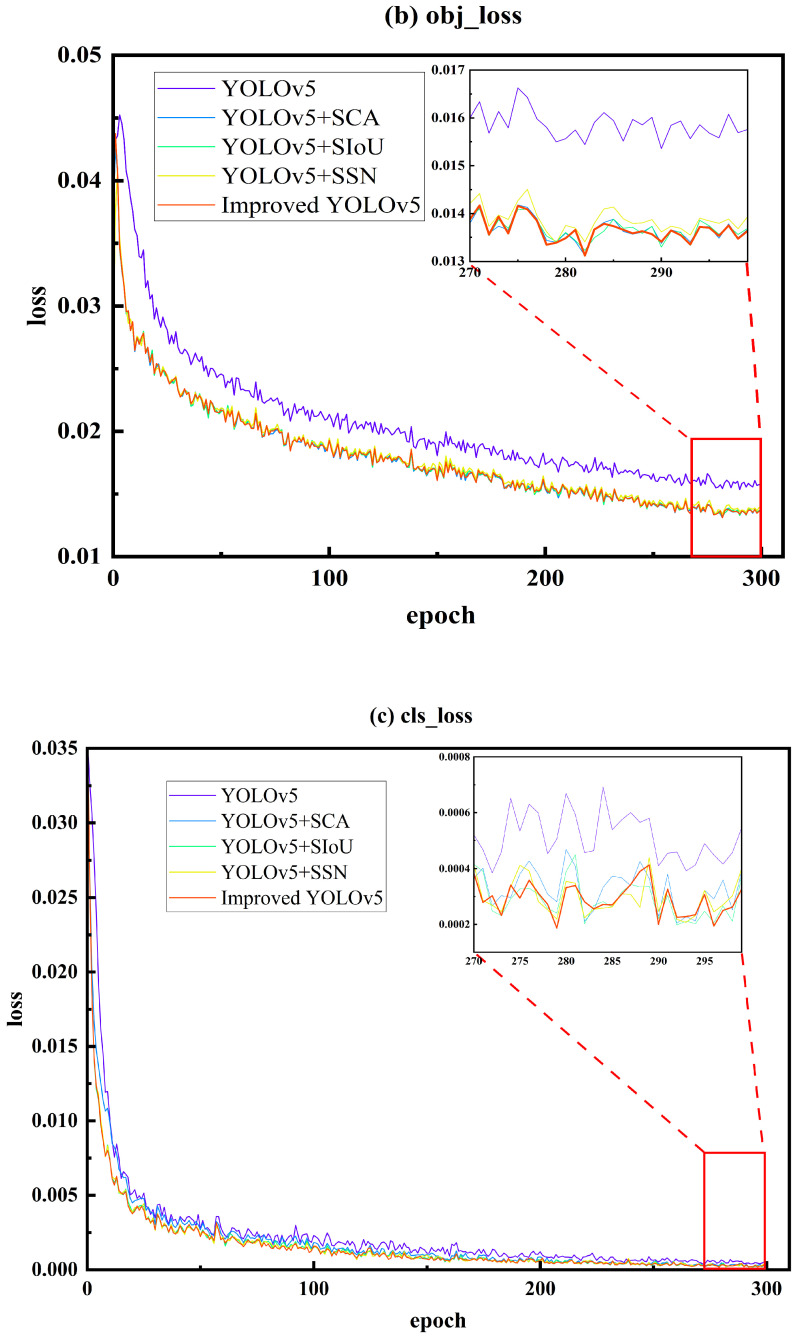
Training Loss Curve Graph ((**a**) box_loss, (**b**) obj_loss, and (**c**) cls_loss).

As shown in Figure 7, the top-down sequence represents the localization loss, object confidence loss, and classification loss. The experiment was conducted for 300 iterations, and after the training, the three types of losses gradually decreased, all reaching the minimum value at the last epoch.

Based on Figure 7, it can be observed that the model exhibited a faster fitting speed in the first 50 iterations and tended to stabilize around 300 iterations, indicating the convergence of the model. In terms of the localization loss, the order of the loss magnitude from smallest to largest was as follows: Improved YOLOv5 < YOLOv5 + SSN < YOLOv5 + SCA < YOLOv5 + SIoU < YOLOv5. The proposed model achieved the lowest loss, indicating the smallest error between the predicted and annotated bounding boxes. YOLOv5 + SSN also exhibited a relatively low loss due to the better generalization regression method of SSN compared to the initial NMS. As for the object confidence loss, compared to the base model, YOLOv5, the proposed model achieved the lowest loss of 0.01364, indicating the smallest cross-entropy loss between the probability of the predicted boxes containing objects and the probability of the annotated boxes containing objects, which represented the highest probability of objects being contained within the bounding boxes. In terms of the category loss, the proposed model had the lowest loss, followed by YOLOv5 + SIoU. Similarly, the improved models had lower losses compared to YOLOv5, indicating a higher probability of containing target categories in the proposed model and improved model performance.

**Figure 8:**
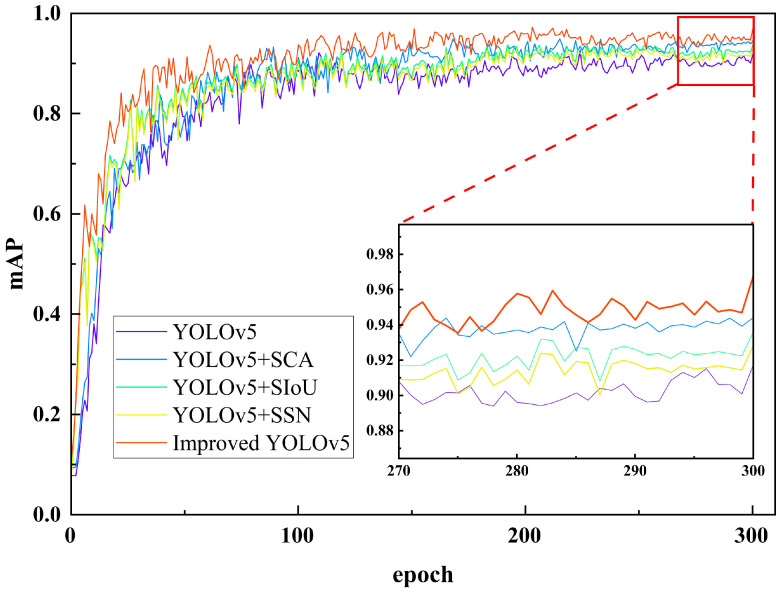
Comparison of Training mAP Graph.

From Figure 8, it can be seen that the model had a faster fitting speed in the first 50 iterations, and tended to be stable when it reached 300 iterations. The model basically converged at this point. For the localization loss, the loss from small to large was: Improved YOLOv5 < YOLOv5 + SSN < YOLOv5 + SCA < YOLOv5 + SIoU < YOLOv5. Among them, the loss of this paper’s model was the lowest, indicating that the error between the predicted box and the calibrated box was the smallest. The loss value of YOLOv5 + SSN was also low, because SSN had a more general regression method than the initial NMS. For the target confidence loss, the loss value of this paper’s model reached the lowest, at 0.01364. The results of the various models compared with the basic model YOLOv5 showed that the improved network structure had a better accuracy than the original model. For the category loss, the loss value of this paper’s model was the lowest, followed by YOLOv5 + SIoU. Similarly, the loss value of the improved model was lower than that of YOLOv5, indicating that the performance was improved.

#### 4.4.2. Test Results and Analysis

**Figure 9:**
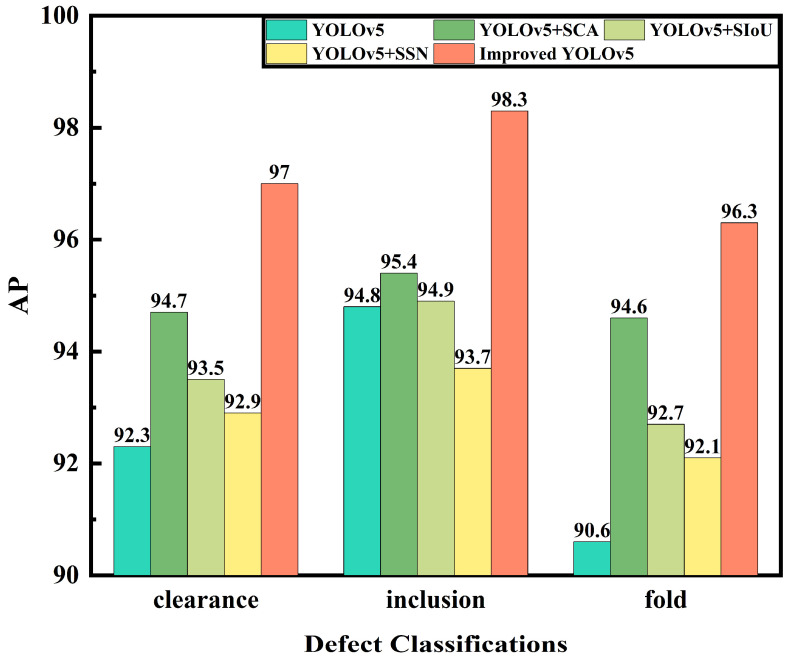
Comparison of Detection AP for Different Types of Surface Defects across the Given Models.

To verify the detection effect of the model, five models were used to detect and recognize 120 composite material winding surface defect images in the test set. Figure 9 shows the AP curves of each category obtained after the detection. It can be observed that, compared to the other two defects, the detection accuracy of inclusions was higher, and the AP curve tilts to the right. Among the equivalent models, the detection of clearance defects was also higher than that of folded defects, because the color characteristics of the inclusion defects were more obvious and the number of inclusion defects in the dataset was relatively large. At the same time, the features of the clearance defects generated during the composite material winding process were obvious, usually spanning the beginning and end of an image.

Among them, the YOLOv5 + SSN model had a higher detection accuracy for clearance and folded defects than the original YOLOv5, indicating an improvement in the regression box selection method by Soft-SIoU-NMS for overlapping targets. The YOLOv5 + SIoU model improved the detection accuracy of defect targets by improving the loss function, and its detection AP for three types of defects exceeded that of the YOLOv5 model. However, there was a class imbalance in the detection accuracy, with a higher accuracy for inclusion defects and a lower accuracy for the other two defects, indicating that this improvement still needs to focus more on feature attention.

The YOLOv5 + SCA model had a high detection AP for all three types of defects, and the accuracy of the detection categories was relatively balanced. Its detection AP for inclusion defects was the highest, reaching 95.4%, and the difference in the detection AP between the other two defects was not significant, which were 94.7% and 94.6%, respectively. Introducing the attention mechanism enhanced the model’s feature extraction and learning ability and improved its detection accuracy.

This paper’s model is YOLOv5 + Separate-CA + SIoU + Soft-SIoU-NMS, which integrated the three improvements and improved the AP of all three types of defects, which were 97.0%, 98.3%, and 96.3%, respectively. Compared to YOLOv5, it increased the detection AP by 4.7%, 3.5%, and 5.7%, respectively, and the detection of each class of defect was more balanced.

**Figure 10:**
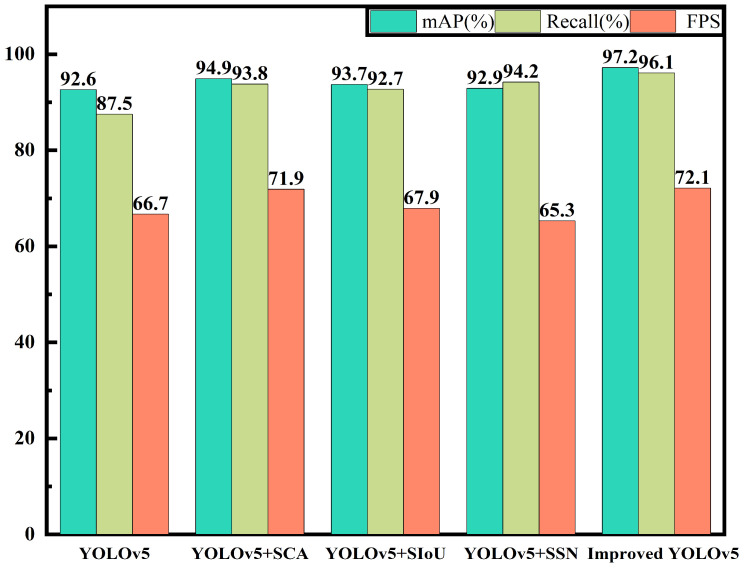
Performance Evaluation of Different Models on the Test Dataset.

Figure 10 shows the performance evaluation of the different models on the test set, including mAP, Recall, and FPS. Experiment 1 was the original YOLOv5 algorithm, with an mAP of 92.6%, a recall rate of 87.5%, and an FPS of 66.7, with an overall mediocre performance. Experiment 2 embedded the SCA module in the main network of YOLOv5. Since the module realizes long-range dependency in the spatial direction and enhances position information, it improved the feature extraction ability. Compared to the YOLOv5 model, its mAP value increased by 2.3% and its recall rate increased by 6.3%. Meanwhile, because the module was inserted using interval embedding, the detection speed also increased, with an FPS of 71.9. Experiment 3 modified the regression box loss function to SIoU. Although the network structure remained unchanged, the introduction of the angle loss improved the accuracy of the back-propagation. The mAP value increased by 1.1% compared to YOLOv5 to a final value of 93.7%, with the recall rate being increased to 92.7%. It had little effect on the FPS. Experiment 4 replaced the original NMS box selection method with Soft-SIoU-NMS. The mAP increased by 0.3 percentage points, but the recall rate increased significantly from 87.5% to 94.2%. However, due to computational constraints, the FPS decreased from 66.7 to 65.3, equivalent to detecting 1.4 fewer images per second. Experiment 5 was the improvement set in this paper, which integrated the improvements in experiments 2, 3, and 4. The improved network had an mAP of 97.2%, a recall rate of 96.1%, and an FPS of 72.1. This shows that the improved YOLOv5 algorithm in this paper not only effectively improved the detection accuracy, but also improved the detection speed. It was more accurate for identifying the surface defects of complex or small composite materials, and had the best overall performance.

#### 4.4.3. Application Results and Analysis

**Figure 11:**
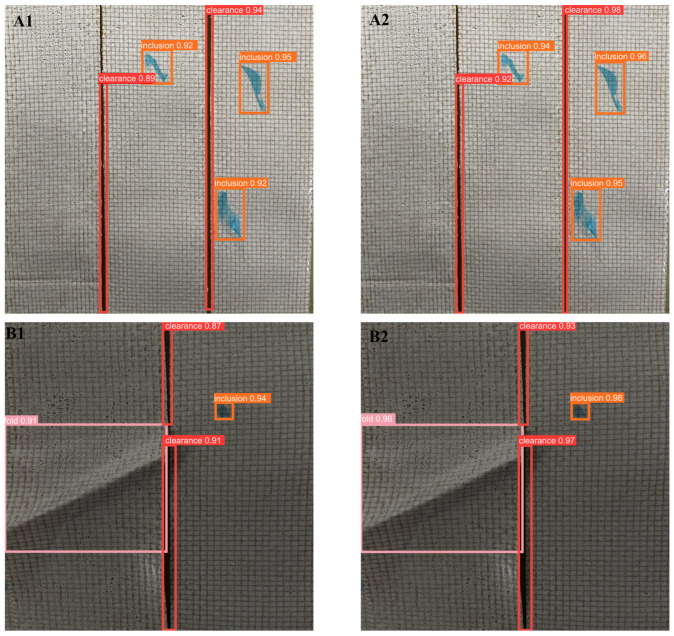

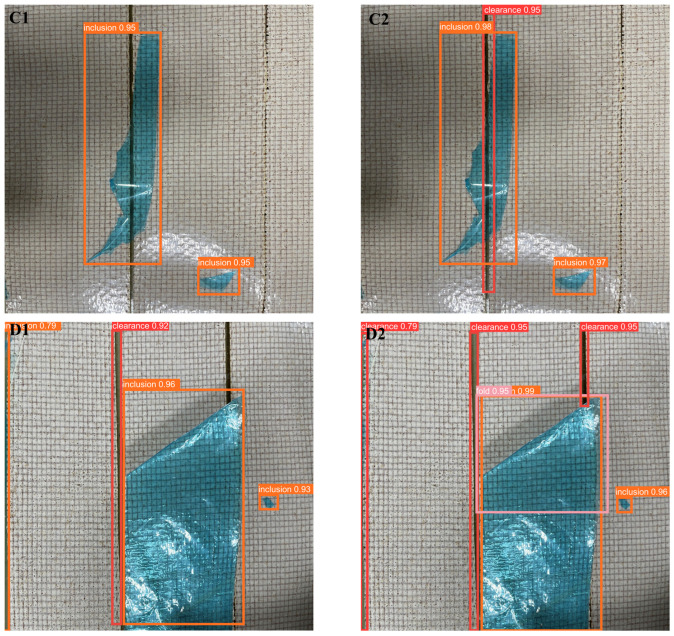

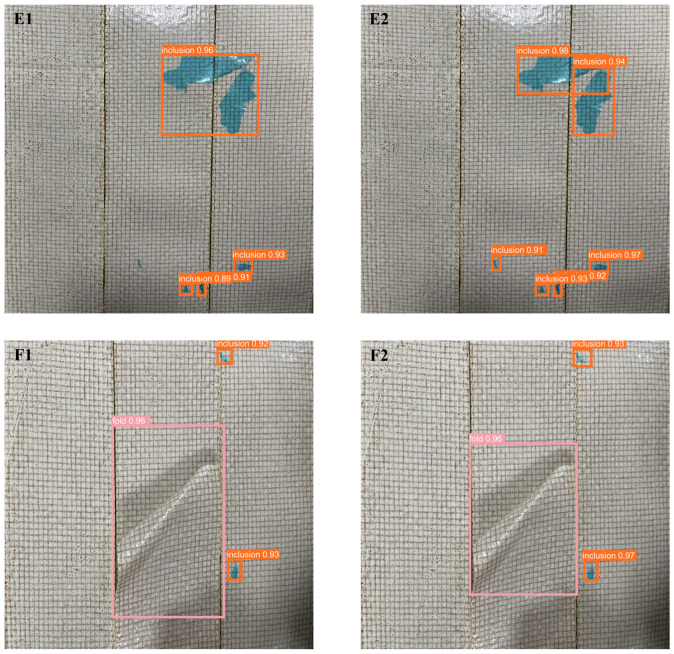
Detection performance of YOLOv5 on video streams before and after improvement (YOLOv5: (**A1**–**F1**), Improved YOLOv5: (**A2**–**F2**)).

This detection model needed to be applied in the actual composite material laying and winding site. Therefore, the detection effect of the model was verified by detecting actual images and comparing YOLOv5 with the improved model in this paper. In the composite material winding site, the winding process was photographed using an industrial camera. The original YOLOv5 model and the model in this paper were used for video detection. Some detection images from the video were captured, and the recognition effect is shown in Figure 11. Considering the possibility of low-confidence defects being filtered out, which would lead to incomplete and inaccurate comparisons, this article did not set a threshold for detection and comparison. In practical application, the determination of the threshold size needs to be initially attempted based on the results of the model testing for setting the confidence threshold, and then continuously fine-tuned during long-term actual detection processes, in order to achieve the best detection efficiency.

From Figure 11, it can be seen that the left side A1~F1 shows the detection results using YOLOv5 before improvement, and A2~F2 shows the detection results using the improved model proposed in this paper. The detection capability of the improved YOLOv5 algorithm was significantly enhanced. Specifically, in A1 and A2 of Figure 11, the image brightness was normal, and both algorithms detected all defects. However, the improved YOLOv5 had a much higher confidence in detecting targets than the original YOLOv5. In the B images, which were captured when the light source was blocked during the winding process, the overall image was darker, but both detection models were not affected by the brightness and accurately detected all the defects. Similarly, the improved YOLOv5 had a greater confidence. In C1 and C2, a large blue inclusion defect partly covered the clearance defect. The original YOLOv5 only detected the inclusion defect, but not the clearance defect. The improved YOLOv5 not only detected all the defects, but also had a higher confidence. In the image D, there were many defects, and the folded defect and inclusion defect overlapped. The original YOLOv5 had instances of false detection and missing detection. It detected the clearance defect on the left side of the image as an inclusion defect and missed the clearance defect on the right side. In contrast, the improved YOLOv5 algorithm effectively detected all the defects. Comparing E1 and E2 shows that the improved YOLOv5 algorithm not only had a higher confidence in detecting targets, but also detected small defects that were undetected in E1. Moreover, for dense inclusion defects, the original YOLOv5 detected both defects as one, while the proposed algorithm distinguished them, resulting in more precise detection results. In F1 and F2, both detection algorithms detected all defects, and the confidence in detecting the folded defect was the same. However, by examining the figure, it can be seen that the bounding box of the improved YOLOv5 was more accurate.

## 5. Conclusions

An improved defect detection algorithm for the automated laying and winding process of composite materials was proposed in this paper, which combined machine vision and deep learning. The improved YOLOv5 algorithm, which integrated the Separate CA + SIoU + Soft-SIoU-NMS module, achieved accuracy improvements of 4.7%, 3.5%, and 5.7%, respectively, for the three types of defects: clearances, inclusions, and folds. The mAP was also improved by 4.6%, and the FPS was increased by 5.4% compared to the baseline algorithm. These improvements demonstrate the positive impact of the improved module on the model, and a further accuracy improvement was achieved under the condition of satisfying the detection speed. The improved YOLOv5 model had a greater accuracy and met the requirements of detection speed in industrial scenes, making it suitable for the defect detection of composite material automated laying and winding processes. However, the improved algorithm only worked for detecting clearances, inclusions, and folds, and could not detect defects such as resin-rich regions. The next focus of the research is the detection of such defects.

## Figures and Tables

**Table 1:** Experimental Software and Hardware Configuration.

Name	Configuration/Version
Operating System	Windows 10 × 64
CPU	Intel(R) Core(TM) i7-10875H
GPU	NVIDIA GeForce GTX 1650 Ti
Memory	32 GB
Graphics memory	4 GB
IDE	Pycharm 2020.1
Deep Learning Framework	Pytorch 1.10.1
CUDA	CUDA 10.2.89
cudnn	cuDNN 8.3.3
PythonVersion	Python 3.9

**Table 2:** Model Training Parameter Settings.

Parameter	Setting
Initial Learning Rate	0.01
Epoch	300
Batch size	3
Momentum Size	0.937
Weight Decay Coefficient	0.0005
Input Image Size	640 × 640
Nc	3
Optimizer	SGD

## Data Availability

Not applicable.
